# Influence of Nanoclay Content on Cement Matrix for Oil Wells Subjected to Cyclic Steam Injection

**DOI:** 10.3390/ma12091452

**Published:** 2019-05-05

**Authors:** Ahmed Abdulhamid Mahmoud, Salaheldin Elkatatny, Abdulmalek Ahmed, Rahul Gajbhiye

**Affiliations:** College of Petroleum Engineering and Geosciences, King Fahd University of Petroleum & Minerals, 31261 Dhahran, Saudi Arabia; g201205160@kfupm.edu.sa (A.A.M.); g201080240@kfupm.edu.sa (A.A.); rahulg@kfupm.edu.sa (R.G.)

**Keywords:** cement retrogression resistance, Saudi class G cement, oil well cement, nanoclay particles, cyclic steam injection

## Abstract

High-temperature conditions drastically compromise the physical properties of cement, especially, its strengths. In this work, the influence of adding nanoclay (NC) particles to Saudi class G oil well cement (OWC) strength retrogression resistance under high-temperature condition (300 °C) is evaluated. Six cement slurries with different concentrations of silica flour (SF) and NC were prepared and tested under conditions of 38 °C and 300 °C for different time periods (7 and 28 days) of curing. The changes in the cement matrix compressive and tensile strengths, permeability, loss in the absorbed water, and the cement slurry rheology were evaluated as a function of NC content and temperature, the changes in the structure of the cement surfaces were investigated through the optical microscope. The results revealed that the use of NC (up to 3% by weight of cement (BWOC)) can prevent the OWC deterioration under extremely high-temperature conditions. Incorporating more than 3% of NC severely damaged the cement matrix microstructure due to the agglomeration of the nanoparticles. Incorporation of NC particles increased all the cement slurry rheological properties.

## 1. Introduction

The main functions of the primary cementing in oil wells is to provide the desired mechanical stability, prevent any communication between the drilled formations by preventing the cement/formation annulus being present, protect the casing from being contacted by the formation of corrosive fluids so no microcracks should be present in the cement sheath, and prevent the fluids from flowing up toward the surface through the cement/formation annulus, microcracks in the cement sheath, and/or casing/cement annulus. In these kinds of operation, the wellbore/casing annular space is filled by one or more cement slurries which must form strong enough and durable cement matrix to meet the needed isolation efficiency throughout the well life without the need for any minimal corrective interventions. To achieve these goals, the cementing project must be well evaluated, discussed, planned and executed [1,2].

One of the most essential parameters to be considered during the design of the cement slurry is the temperature to which the cement sheath will be subjected. The effect of the temperature on both the liquid phase when the slurry is pumped through the casing/wellbore annular space, and on the solid phase (hydrate cement) when the cement matrix is required to meet the physical and chemical properties considered when the cement program is designed, was earlier addressed by different studies [2,3,4,5,6,7].

Alteration of the mechanical properties of hydrated Portland cement is expected at high-temperature conditions, since the elevated temperature adversely effects on the cement hydrated products [6,8,9,10,11,12,13,14,15]. The conditions of elevated temperature are expected in deep wells, geothermal wells, and wells subjected to steam injection for enhanced oil recovery (EOR) applications, since the injected steam is able to decrease the oil viscosity, and hence, increase its mobility and finally enhance the oil production [16]. Currently, thermal oil recovery is a widely used technique around the world to produce heavy oil in many projects, examples are: (a) Kern Field in California, USA; (b) the Athabasca Oil Sands in Alberta, Canada; (c) Mene Grande in Venezuela; (d) Northeast Region in Brazil; and (e) the Surplacul de Barcau Field in Romania, [17,18,19].

Portland cement experiences major chemical and microstructural transformations under high-temperature conditions (when the temperature exceeds 110 °C), such a phenomenon is known as strength retrogression which intensifies as long as the temperature increases beyond 110 °C [3,20,21]. During the strength retrogression transformation, calcium-rich products are formed in the cement matrix, which will result in an increase of the matrix permeability and a deterioration of its mechanical properties.

Several early studies confirmed that when the temperature exceeds 110 °C, and based on the temperature value, the addition of the silica (SiO_2_) particles (usually greater than 30% by weight of cement (BWOC)) into the cement could considerably enhance the cement resistance to the strength retrogression [7]. Interaction of the reactive SiO_2_ particles with portlandite phase (Ca(OH)_2_ or CH), which is one of the main products of the cement hydration process, usually lead to formation of another stable phase of calcium silicate hydrated (C-S-H) species like tobermorite, trucostite, and xonotlite which are able to enhance the cement matrix mechanical properties [20,21,22,23,24]. Anti-retrogression agents are materials rich in silica such as silica sand and silica flour, which are mostly added to the oil well cement (OWC).

Table 1 summarizes the reaction velocities of the silicate (pozzolan) cement hydration and the reaction of CH with the slow pozzolans. This table shows that the reaction of the slow pozzolans such as nanoclay (NC) with CH is a slow reaction compared to the other reactions happen during the cement hydration process.

Costa et al. [6] examined the use of silica particles to enhance the oil wells cement resistance to the high-temperature conditions. As a result, they reported that the optimum concentration of silica particles that could considerably enhance the cement resistance to the high-temperature conditions of 300 °C is 35% BWOC.

The NC particles are currently used in the cement industry to enhance the strength of the cement matrix because of their ability to fill the capillary microporous of the matrix due to their considerably small size, the property which will increase the density of the solidified cement. Sanjuán et al. [26] reported that the decrease in the particle size of the pozzolanic materials (such materials like NC and silica fume) considerably improved the cement strength. Wang [7] studied the effect of replacing the ordinary Portland cement (OPC) with 0.1 to 0.5% of the NC particles on the concrete strength at a variable temperature from 25 to 1000 °C. As a result, Wang [7] reported that the NC particles are able to enhance the concrete compressive strength with the increase of the temperature up to 300 °C; a significant decrease in the concrete compressive strength was noticed when the temperature is between 440 to 580 °C, and the compressive strength of the concrete reduced to about 10% of the original strength when the temperature reached 1000 °C.

The NC particles are characterized by their extremely large surface area and very small particle size, such properties accelerate the reactions happen during the cement hydration process, and hence, more secondary C-S-H gel will be produced which will effectively fill the cement matrix capillary pores [27,28,29], and thus, densifying the microstructure of the matrix.

The aim of this study is to evaluate the applicability of adding different percentages of NC with and without 35% BWOC of silica flour (SF) particles to Saudi class G oil well cement (OWC) to enhance its compressive strength under high-temperature conditions (300 °C) encountered in geothermal environments and in wells experience cyclic steam injection.

## 2. Methodology

In this study, exposure of the cement samples to high temperature (300 °C) was considered and followed by the analysis of the mechanical behavior of the cement samples.

### 2.1. Materials

The slurries considered in this study were formulated using Saudi class G cement, silica flour, friction reducer additive, different concentrations of the NC particles, and deionized water. The cement slurries compositions are summarized in Table 2.

Saudi class G cement with the specific gravity (SG) of 3.15 and the chemical composition summarized in Table 3 as characterized by X-ray fluorescence (XRF) technique, and the phases composition summarized in Table 4 was used in this study, the phase composition of Saudi Class G cement summarized in: 

Table 4 indicates that this type of cement is a high sulfate resistant.

**Figure 1:**
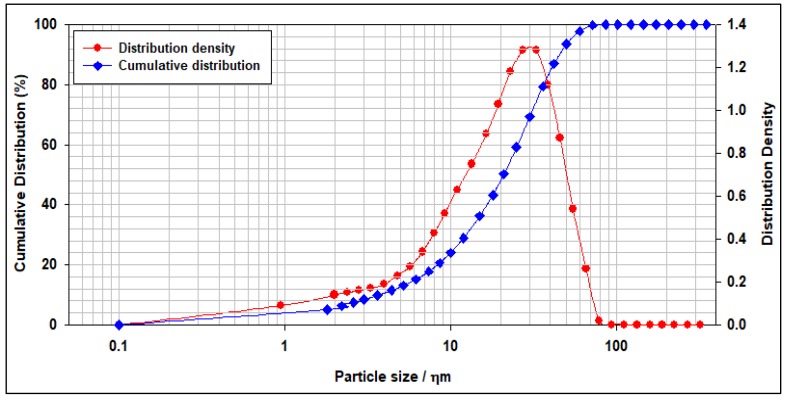
The particles size distribution of Saudi Class G cement.

Figure 1 shows the particles size distribution (PSD) for the Saudi Class G cement used in this study. As shown in Figure 1 more than half of the cement particles are less than 21.27 µm, and about 90% of the cement particles are less than 47.18 µm. 

The silica flour is a material which is composed of more than 99% SiO_2_ [5,30] and has an SG of 2.64 g/cm^3^, the friction reducer (CFR-3) is a special friction reducer provided by a service company. The deionized water has an SG of 1.0. The NC particles used in this study are modified montmorillonite nanoclays which is organically modified by cation exchange reaction to transform it to more hydrophobic state according to the procedures explained by Rahman et al. [31], the NC particles SG is 1.98.

**Figure 2:**
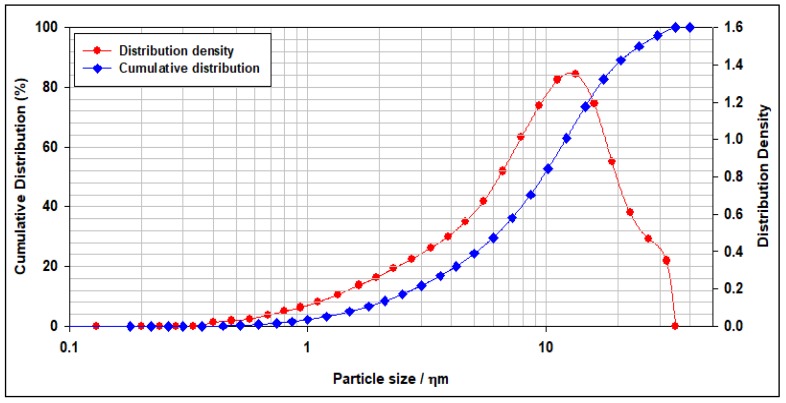
The particles size distribution of the nanoclay (NC) particles.

Figure 2 shows the PSD for the modified montmorillonite NC particles used in this study. As indicated in Figure 2 more than half of the cement particles are less than 9.69 µm, and about 90% of the cement particles are less than 21.46 µm.

### 2.2. Samples Preparation and Curing

Cement slurries with a density of around 1.97 g/cm^3^ (16.44 ppg) were prepared and tested following the American Petroleum Institute procedure [32]. Six slurries with the composition in Table 2 were prepared. The first slurry (S0NC0) was prepared without adding SF or NC particles to be considered as the base case. In the second formulation (S35NC0), 35% BWOC of SF was added, since the 35% is considered as the optimum silica content for applications of 300 °C temperature [6], so this formulation (S35NC0) will be compared with the next formulations which contain the same silica content (i.e., 35%BWOC) and different concentrations of NC particles. S35NC1, S35NC2, S35NC3 and S35NC4 samples in Table 2 denote the cement slurries incorporating 1, 2, 3, and 4% BWOC of the NC, respectively.

After preparation, some of the slurries were poured into 50.8 mm edge metallic cubical molds for compressive strength testing and the remaining poured into cylindrical molds of 38.1 mm in diameter and 22.9 mm in length for tensile strength testing, permeability measurements, and water loss testing. The molds were then submerged in a water bath at 38 °C for a specific time. All the formulations in Table 2 were evaluated in two different scenarios. The low temperature of which (38 °C) was used as a reference, and the high temperature of 300 °C which was used to represent the average temperature for wells to undergo cyclic steam injection for EOR. In the case of the low temperature, the samples were submerged into the water bath for 7 and 28 days before testing for the compressive and tensile strengths, and permeability. On the other hand, other samples were kept into the water bath at the low temperature of 38 °C for 4 and 25 days and then subjected to the high temperature of 300 °C and 20.68 MPa for more three days to represent one cycle of steam injection condition, after that these samples were tested for the same properties evaluated in the case of the low-temperature scenario. Table 5 summarizes the curing conditions for the different scenarios considered in this study.

### 2.3. Compressive Strength Measurement

Compressive strength of the samples was evaluated based on the API procedure [32]. For each temperature condition (scenarios in Table 5), and for all compositions under study (Table 2) after 7 and 28 days, three cubical samples with 50.8 mm edge were used to evaluate the cement matrix compressive strength. The compressive strength of every specific composition at a specific temperature condition was calculated based on the average strength of the three tested samples.

### 2.4. Permeability Measurement

Gas permeability of the different cement matrix compositions considered in this study was measured on cylindrical samples of 38.1 mm in diameter and 22.9 mm in length, the samples permeability was calculated using Hagen-Poiseuille law which is a commonly used law to calculate the permeability for a laminar flow under steady-state conditions of a compressible fluid through a porous material composed of a network of small capillary pores, the permeability was measured according the procedures explained earlier by Sanjuán and Muñoz-Martialay [33].

### 2.5. Tensile Strength Measurement

Cylindrical samples of 38.1 mm in diameter and 22.9 mm in length were prepared for the purpose of tensile strength testing. The indirect tensile strength testing method (Brazilian test) was used to measure the maximum load that the sample could resist before falling under tension. Then Equation (1) was used to calculate the tensile strength of the sample.

(1) $$\sigma_{t}=\frac{2P}{\pi dl}$$

where *σ_t_* denotes the Brazilian tensile strength in (MPa), *P* is the failure load in (N), *d* and *l* are the cement sample diameter and length, respectively, both are in (mm).

Three cylindrical samples were used to evaluate the cement matrix tensile strength for every specific composition at a specific temperature condition. The tensile strength was then calculated based on the average tensile strength of the three tested samples.

### 2.6. Non-Evaporable Water Content

The samples mass loss due to exposure to a high temperature was investigated, cylindrical samples of 38.1 mm in diameter and 22.9 mm in length were aged for 4 and 25 days at 38 °C, then they were firstly oven dried at 105 °C for 2 h, after this time there was no more water evaporation at 105 °C, the samples were then weighted before and after being subjected to the high temperature of 300 °C for 3 days. The loss in the weight after exposing the samples to (300 °C) is related to the loss in the amount of the water absorbed by NC particles.

### 2.7. Optical Microscope Images

Cubical samples with 50.8 mm edge were prepared for the purpose of imaging. After samples curing at 38 °C for 7 days they were cured at 300 °C for three more days, then a small cubical slice with the dimensions of 5 × 5 × 20 mm^3^ was cut exactly from the center of each cement cube for the purpose of imaging through optical microscope to study the change in the pore structure of the cement matrix as a function of the SF and NC concentrations. The cement slices were polished prior to the microscope imaging.

### 2.8. Rheology

Addition of NC particles is known to considerably alter the cement rheology as reported earlier by many authors [34,35]. Alteration of rheological characteristics for all cement slurries considered in this study was evaluated to address the effect of the NC particles on the cement rheology. The 10-s and 10-min gel strengths (GS), yield point (YP), and plastic viscosity (PV) were evaluated for all of the cement slurries under study.

## 3. Results and Discussion

### 3.1. Compressive Strength Results

**Figure 3:**
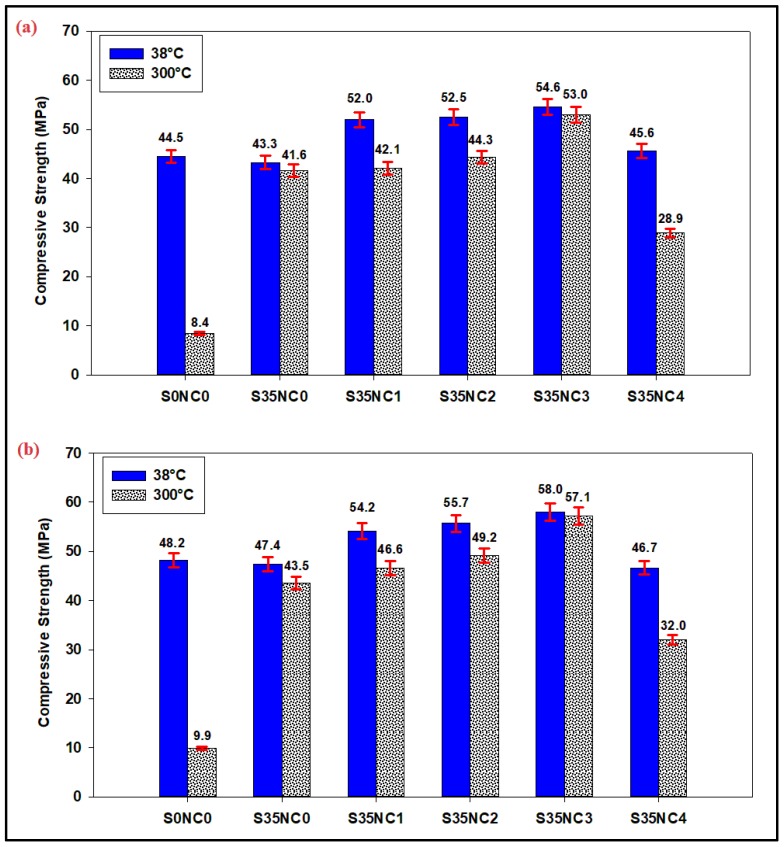
The compressive strength of the samples cured at 38 °C and 300 °C after (**a**) 7 days (**b**) 28 days.

In this section, the effect of temperature on the strength retrogression for the different slurries under consideration is evaluated; since the cement strength retrogression is expected to occur when the well temperature exceeds 110 °C [21,36]. Figure 3 compares the change in the strength for all the cement formulations with and without subjecting the cement samples to 300 °C. The base sample (S0NC0) which has zero SF and NC content lost 81.12% and 79.46% of its original strength after 7 and 28 days of curing, respectively, when exposed to 300 °C condition. The strength of the sample S0NC0 after 7 days decreased from 44.5 to 8.4 MPa and after 28 days decreased from 48.2 to 9.9 MPa when undergoes a condition similar to one cycle of steam injection.

When 35% BWOC of the SF only is added to the cement slurry (sample S35NC0), a considerable increase in the cement resistance to the strength retrogression at a temperature of 300 °C was observed after 7 and 28 days, as shown in Figure 3. The strength of the sample S35NC0 exposed to 300 °C after 7 days is 41.6 MPa compared to 8.4 Mpa for the base sample (S0NC0), and after 28 days the strength was 43.5 Mpa when the sample subjected to one steam injection cycle condition (Figure 3), which is also very high compared to that of sample S0NC0 (9.9 Mpa), this is attributed to the fact that during the pozzolanic reaction, interaction of SiO_2_ particles with the CH leads to the formation of more stable C-S-H products during the hydration process [25].

Addition of NC particles up to 3% BWOC to the slurry containing 35% BWOC of SF enhanced the cement strength no matter the temperature used. At low temperature (38 °C), 3% of the NC were able to increase the strength of the cement by 26.10%, after 7 days and by 22.36% after 28 days over that obtained by using 35% of SF only (sample S35NC0), this could be explained as a result of the quick reaction of the NC particles which are characterized by the extremely large surface area and its amorphous state (small particle size) with the free lime during the hydration process, and hence, more secondary C-S-H gel will be produced which will effectively fill the cement matrix capillary pores [27,28,29], and thus, densify the microstructure of the matrix.

At high temperature (300 °C), adding 1, 2, or 3% BWOC of the NC particles which are stable at a temperature conditions to the slurry with 35%BWOC of SF was able to enhance the strength retrogression resistance compared to the sample S35NC0 which has only SF particles after both 7 and 28 days of curing (Figure 3). The strength increased to 53.0 MPa after 7 days when 3% NC is added compared to 43.3 MPa for sample S35NC0, and after 28 days the strengths of 57.1 and 43.5 MPa were observed for S35NC3 and S35NC0, respectively.

In both cases of low and high temperatures, addition of 4% BWOC of NC (sample S35NC4) lead to deterioration of the cement strength. The compressive strength of sample S35NC4 after 7 days in the case of high-temperature scenario becomes 28.9 MPa which is less than that of the sample with zero nanoclay (41.6 MPa), and after 28 days it becomes 32.0 MPa, which is again less than that of the sample with zero nanoclay (43.5 MPa). This result is attributed to the fact that the use of high concentration of nanoparticles (i.e., more than 3%) will lead to nanoparticle agglomeration in the mixes. Formation of weak zones will occur because of these aggregations, which in turn prevents homogenous hydrate formations to form, as a result, the strength of the cement will deteriorate at low-temperature conditions and the strength retrogression resistance will drastically decrease when the cement matrix exposed to high-temperature conditions [37,38].

### 3.2. Tensile Strength Results

**Figure 4:**
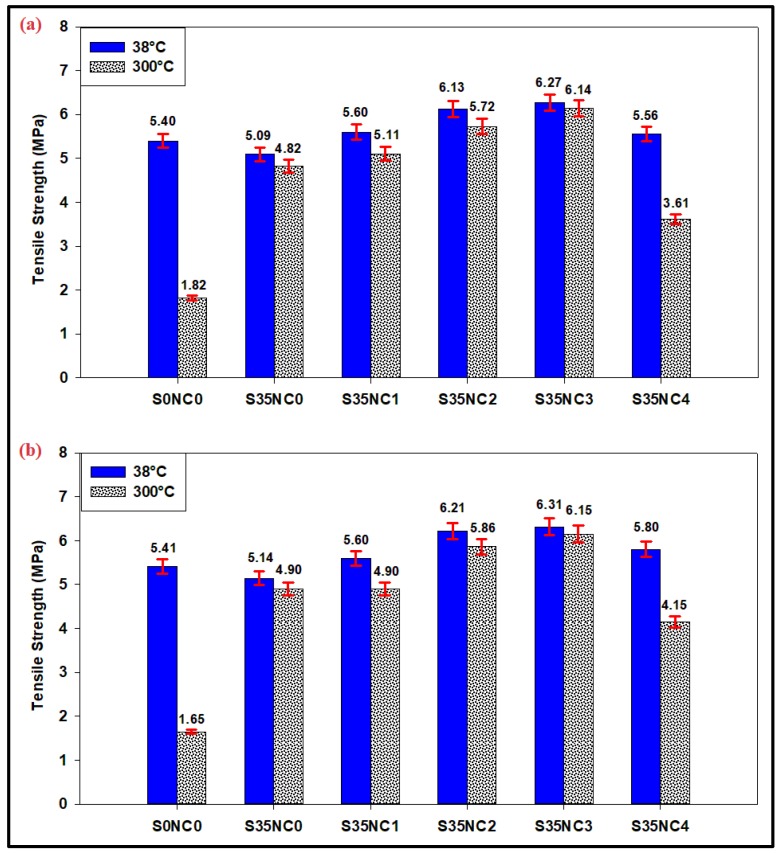
The tensile strength of the samples cured at 38 °C and 300 °C after (**a**) 7 days (**b**) 28 days.

The tensile strengths of the cement samples are presented in Figure 4, sample S0NC0 loss 66.30% and 69.50% of its tensile strength after 7 (Figure 4a) and 28 days (Figure 4b), respectively, when subjected to the high-temperature scenario. Including 35% of SF particles (sample S35NC0) considerably enhanced the cement resistance to failure under tensile force and high-temperature conditions, because of the formation of stable forms of C-S-H during the pozzolanic reaction between the reactive SiO_2_ and CH (Table 1) [20]. Incorporating NC (up to 3% BWOC) into the cement formulation improved its tensile strength linearly under high-temperature conditions, which is a result of the accelerated hydration process.

At high-temperature conditions, sample S35NC3 with 3% NC and 35% SF content has tensile strengths of 6.14 (Figure 4a) and 6.15 MPa (Figure 4b) after 7 and 28 days, respectively, with an enhancement of 27.39% and 25.51% as compared to sample S35NC0 which contains SF only. The cement resistance to tension declined when 4% of NC is added, since the system overloaded with the nanoparticle, and hence, an irregular microstructure is expected to be developed. Figure 4 shows that sample S35NC4 tensile strengths at low-temperature conditions are 5.56 and 5.80 MPa after 7 and 28 days, respectively, at high-temperature conditions the tensile strengths of sample S35NC4 decreased by 25.10% and 15.30% compared to sample S35NC0 after 7 and 28 days respectively.

### 3.3. Permeability Measurement Results

**Figure 5:**
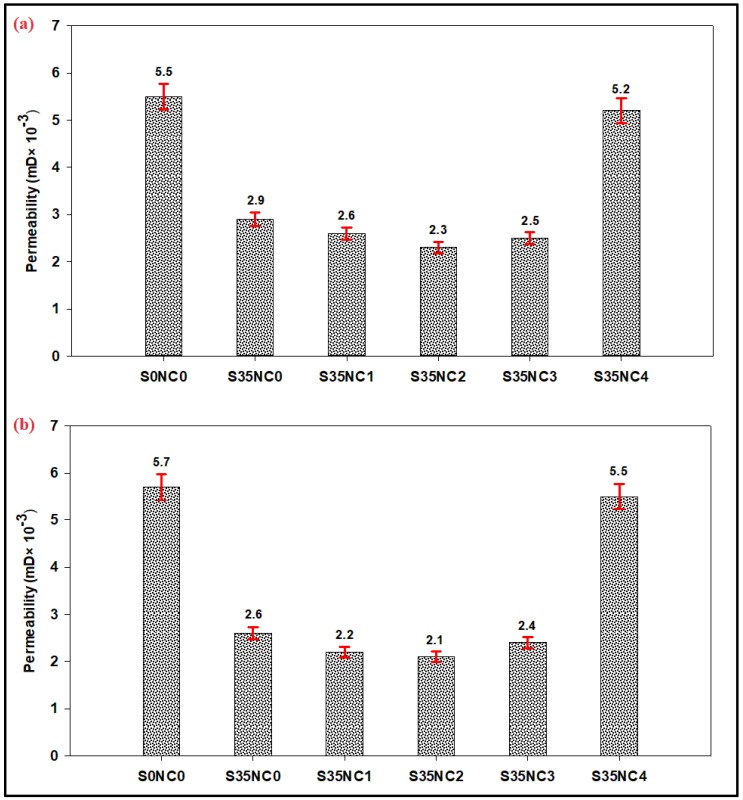
The permeability of the samples after experiencing a condition similar to one cycle of steam injection at 300 °C for 3 days after being cured at 38 °C for (**a**) 4 days (**b**) 25 days. The total curing times are (**a**) 7 days and (**b**) 28 days.

Figure 5 summarizes the permeability measurement results for the cement samples exposed to a condition similar to one cycle of steam injection at 300 °C for 3 days. These samples firstly cured at 38 °C using the water bath for 4 days (Figure 5a) and 25 days (Figure 5b) then exposed to a high-temperature condition of 300 °C for three days using a high-pressure and high-temperature (HPHT) curing chamber, the total curing time periods are 7 (Figure 5a) and 28 days (Figure 5b), all cement samples were dried before air permeability measurements as recommended by Sanjuán and Muñoz-Martialay [37].

The base sample (S0NC0) has a permeability of 0.0055 and 0.0057 mD after 7 and 28 days, respectively, as shown in Figure 5. This figure also shows that the addition of 35% SF into the cement formulation (sample S35NC0) was able to decrease the cement permeability by 47.27% after 7 days (Figure 5a) and 54.39% after 28 days (Figure 5b) compared with sample S0NC0. The cement permeability could be decreased more by incorporating NC particles as indicated in Figure 5b, the addition of 2% NC (sample S35NC2) decreased the cement permeability by 20.69% and 19.23% compared to sample S35NC0, which had SF only. When 3% of NC particles were added, the cement permeability slightly increased (compared to the sample with 2% NC) by 0.0002 (Figure 5a) and 0.0003 mD (Figure 5b) after 7 and 28 days, respectively, the reason for this slight increase will be explained in the next section.

Increasing the NC concentration beyond 3% (i.e., 4% of nanoclay for sample S35NC4), as in the case of sample S35NC4, lead to a huge increase in the permeability which increased to 0.0052 and 0.0055 mD after 7 and 28 days, respectively, compared with 0.0025 and 0.0024 mD for sample S35NC3. This huge increase in the permeability is caused by the agglomeration of the nanoparticles. The use of a higher concentration of nanoparticles could lead to particles agglomeration as reported earlier by Shebl et al. [38] and Hakamy et al. [39] which results in an irregular microstructure, and hence, high permeability. Then when sample S35NC4 exposed to high-temperature, induced microcracks caused by the irregular microstructure could be easily formed. The evaporated water from the cement matrix and the loss of the water absorbed by nanoclays at high temperature is also responsible about this increase in the permeability as will be explained in the next section.

Table 6 compares between two permeability values for each cement sample at every curing period. For every curing time (i.e., 7 and 28 days) one sample was cured at 38 °C using the water bath for the whole period while another sample removed and curing at 300 °C during the last three days of curing to represent a condition similar to one cycle of steam injection. Table 6 shows that sample S0NC0 which has no SF or NC experienced a considerable increase in its permeability when exposed to 300 °C during the last three days of curing compared with the same sample cured at low-temperature of 38 °C for the whole curing period. Sample S0NC0 permeability increased by 71.88% and 96.55% after 7 and 28 days, respectively.

When 35% BWOC of SF is added (sample S35NC0), the samples permeability at high-temperature condition decreased by 23.68% and 27.78% after 7 and 28 days, respectively, compared to the same sample when cured at 38 °C for the whole curing period (Table 6). This result is attributed to voids filling up due to matrix expansion as the temperature increases as noticed for concrete mortars saturated with silica particles by Farzadnia et al. [40] and because of transformation of the CH to more stable forms of C-S-H as reported by Heikal et al. [41] and Heikal [42].

It’s noticed in Table 6 that the permeability values for all NC based samples increased when exposed to high-temperature conditions compared with the permeability of the same samples when cured at the low-temperature conditions. This increase is very small for the samples with NC of not more than 3%, for example, sample S35NC3 permeability increased by 0.0013 and 0.0014 mD after 7 and 28 days, respectively, after exposure to 300 °C compared with when sample S35NC3 is cured at low temperature only. Although of this increase all the samples with NC concentrations of not more than 3% still have permeabilities less than the sample S35NC0 which has no NC particles, as indicated in Figure 5 and Table 6. Sample S35NC4 with 4% NC experienced a considerable increase in the permeability by 92.56% and 120% after 7 and 28 days, respectively, when subjected to 300 °C.

### 3.4. Non-Evaporable Water Content and Optical Microscope Images

**Figure 6:**
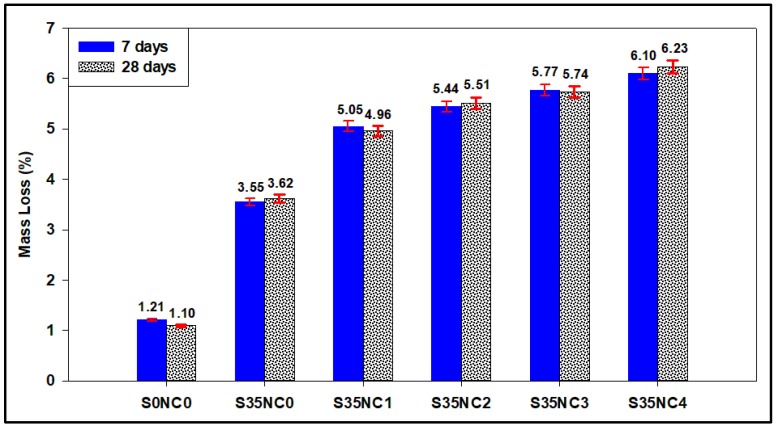
The mass loss after exposing the dried samples to 300 °C for 3 days.

**Figure 7:**
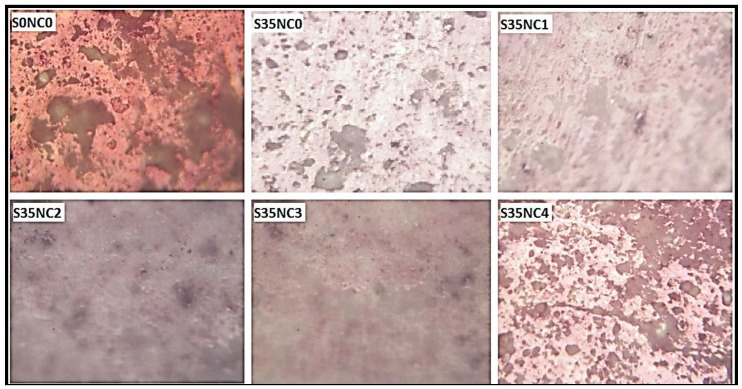
The optical microscopy images of the cement specimens exposed to 300 °C.

Figure 6 summarizes the mass loss of the samples exposed to 300 °C for 3 days after being dried for 2 h at 105 °C. These samples exposed to the high-temperature condition after being aged for 4 and 25 days at low-temperature conditions of 38 °C, so the total aging times are 7 and 28 days (the same second scenario in Table 5). The results in Figure 6 show that the sample with no SF and NC particles lost minor non-evaporable water of 1.21% and 1.10% after 7 and 28 days, respectively. This result confirms that the huge increase in the permeabilities of sample S0NC0 (Table 6) when exposed to a high-temperature condition is related to the matrix damage and not water evaporation, the optical microscope image in Figure 7 shows that sample S0NC0 has a considerable pore spaces after exposure to 300 °C which confirm that the increase in the permeability of this sample is related to the increase in the void spaces of the sample.

Incorporating NC into the cement matrix increased the concentration of the non-evaporable water remains after the samples were dried at 105 °C, which confirms that the increase in the samples S35NC1, S35NC2, and S35NC3 permeabilities after heating the samples at 300 °C compared to the samples cured at 38 °C (Table 6) is related mainly to the evaporation of the water absorbed by the NC particles, Figure 7 shows that these samples have fewer void spaces compared with the samples with no NC, which confirm that the increase in the permeability of these samples is not due to cement matrix damage.

Although sample S35NC4 non-evaporable water content is the highest (6.10% and 6.23% after 7 and 28 days, respectively), this does not confirm that the enhancement in the permeability of this sample when subjected to 300 °C compared with when treated at 38 °C as shown in Table 6 is mainly related to the non-evaporable water when compared with the non-evaporable water content and permeability increase for sample S35NC3, since both samples have high non-evaporable water concentration but the permeability enhancement for the sample S35NC4 is very high compared to sample S35NC3 Table 6. Figure 7 confirms that sample S35NC4 is dominated by microporous which is a result of a non-uniform microstructure of this sample caused by the agglomeration of the NC particles.

### 3.5. Effect on Rheological Parameters

**Figure 8:**
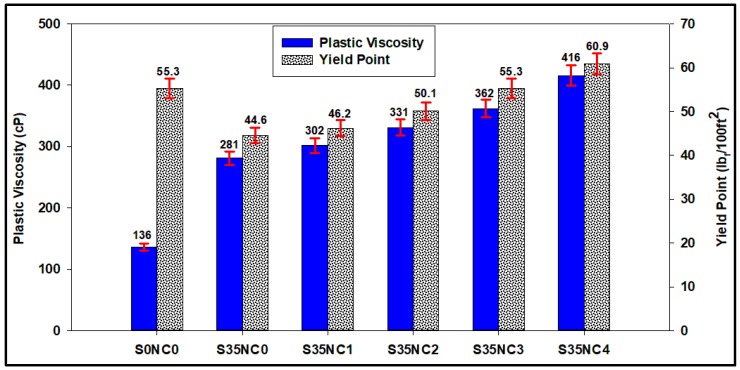
Comparison of the plastic viscosity and yield point for the different cement slurries.

The effect of the NC particles on the rheological characteristics of cement slurry was evaluated. As shown in Figure 8, sample S35NC0 which contain 35% of the SF and no NC particles had PV and YP of 281 cP and 44.6 lb_f_/100 ft^2^, respectively. Incorporating NC into the cement slurries increased both the PV and YP of the slurry. Addition of 1% BWOC of NC to the cement slurry (sample S35NC1) increased the PV and YP of the cement by 7.5% and 3.6%, respectively, compared to sample S35NC0. Both PV and YP of the cement slurries increased with the increase in the NC concentration to reach 416 cP and 60.9 lb_f_/100 ft^2^, respectively, for sample S35NC4 containing 4% BWOC of NC. It is very important to address the change in the PV and YP of the cement slurry; since PV is the main factor controlling the pumpability of the cement slurry from the surface down into the wellbore through the tubing and then up through the annulus between the well casing and the drilled formations, while the increase in the YP of the slurry is required to improve the carrying capacity of the cement slurry. Compared with the base cement slurry, the cement slurries containing NC required high energy to be pumped into the well as indicated by the increase in the PV for the samples containing NC. Addition of the NC particles into the cement slurry also increased its YP, which means prevention of solids segregation and settlement of fine particles while cement is injected to fill the annular space between the drilled formations and the well casing.

**Figure 9:**
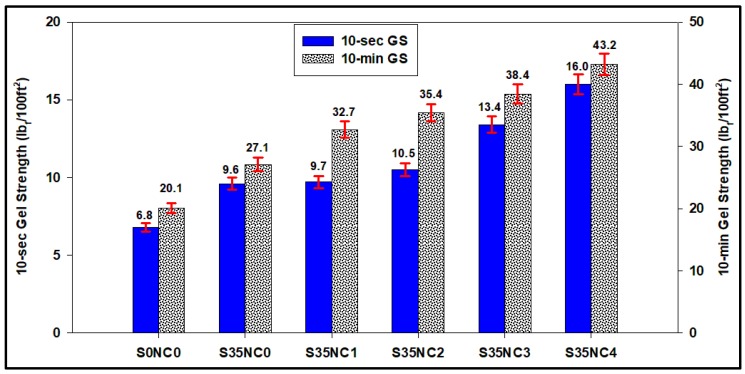
Comparison of the 10-s and 10-min gel strengths for all cement slurries.

Figure 9 compares the 10-s and 10-min GS of the different cement slurries considered in this study. The 10-s and 10-min GS of the slurry S35NC0 with no NC particles are 9.6 and 27.1 lb_f_/100 ft^2^, respectively. Sample S35NC1 with 1% BWOC of NC particles has 10-s and 10-min GS of 9.7 and 32.7 lb_f_/100 ft^2^, respectively. It is clear from Figure 9 that the 10-s and 10-min GS increased as a function of the NC concentration in the cement slurry to reach 16.0 and 43.2 lb_f_/100 ft^2^, respectively. The GS is an important parameter needed while designing the cement slurry since it indicates the ability of the cement to keep the solids particles suspended in the solution when pumping is stopped for any reason, as well as it indicates the energy needed to resume slurry pumping again. Including NC particles into the cement slurry increased its GS, which means prevention of solids segregation and settlement of fine particles when cement circulation is stopped for any reasons, but on the other hand, this may affect the pumpability of the cement especially if the cement circulation is stopped for long time.

## 4. Conclusions

In this study, the effect of incorporating nanoclay particles into the cement matrix compressive and tensile strengths, permeability, and non-evaporable water content at low (38 °C) and high (300 °C) temperature conditions was evaluated. Adding nanoclay particles with concentrations not more than 3% BWOC improved the cement resistance to failure under compression and tension at low and high-temperature conditions as a result of accelerating the cement hydration reaction. It also showed a decrease in the permeability at both low and high temperatures compared to the base cement samples with and without silica flour. When the nanoclays are added with concentrations greater than 3% BWOC, agglomeration of the nanoparticles adversely affected the compressive and tensile strengths at 38 °C and 300 °C; the permeability of the samples was also increased considerably at low temperature due to the microstructure irregularity caused by nanoparticles agglomeration and at high temperature due to samples damage and loss of non-evaporable water. The microscope images confirmed that including NC particles (<3%) improved the pores filling of the cement matrix, while adding more NC (>3%) affected the pore structure of the cement matrix negatively by increasing concentration of the pore space of the cement. Incorporation of NC particles increased all of the cement slurry rheological properties.

## Figures and Tables

**Table 1:** Cement hydration and slow pozzolans reaction description, modified after Bezerra et al. [25].

Reaction	Reaction Velocity
2C_3_S + 6H → C-S-H (61%) + CH	Fast → hours and days
2C_2_S + 4H → C-S-H (82%) + CH	Fast → days
Slow Pozzolans + CH + H → C-S-H (pozzolanic reaction)	Slow → days to months

**Table 2:** Cement slurries compositions.

Slurries	Cement (g)	Silica Flour (%BWOC)	Friction Reducer (%BWOC)	Water (%BWOC)	Nanoclay Particles (%BWOC)
S0NC0	600	0	0.8	44	0
S35NC0	600	35	0.8	44	0
S35NC1	600	35	0.8	44	1
S35NC2	600	35	0.8	44	2
S35NC3	600	35	0.8	44	3
S35NC4	600	35	0.8	44	4

**Table 3:** Saudi class G cement elemental composition, by X-ray fluorescence (XRF) analysis.

Component	Concentration (wt.%)
Silica (SiO_2_)	21.6
Alumina (Al_2_O_3_)	3.30
Iron Oxide (Fe_2_O_3_)	5.99
Calcium Oxide, Total (CaO)	64.2
Magnesium Oxide (MgO)	1.10
Sulphur Trioxide (SO_3_)	2.20
Loss on Ignition	0.90
Insoluble Residue	0.30
Equivalent Alkali (as Na_2_O)	0.41

**Table 4:** Saudi class G cement phase composition.

Component	Concentration (wt.%)
C_3_A	<1
C_3_S	62
C_2_S	15
C_4_AF + 2C_3_A	16

**Table 5:** Curing time procedure.

Temperature	Curing Time
38 °C	7 days in the water bath
	28 days in the water bath
300 °C	7 days, the first 4 days in the water bath and the last 3 days in the curing chamber at 300 °C (one thermal cycle)
	28 days, the first 25 days in the water bath and the last 3 days in the curing chamber at 300 °C (one thermal cycle)

**Table 6:** The permeability changes in (mD × 10^−3^) for the cement samples cured for 7 and 28 days. For each case a sample represents every specimen cured at 38 °C using the water bath for the whole curing time period while another sample removed and cured at 300 °C during the last 3 days of curing period using the HPHT curing chamber to represent a condition similar to one cycle of steam injection.

Sample	7 days		28 days	
	@ 38 °C	Last 3 days @ 300 °C	@ 38 °C	Last 3 days @ 300 °C
S0NC0	3.2	5.5	2.9	5.7
S35NC0	3.8	2.9	3.6	2.6
S35NC1	2.1	2.6	1.9	2.2
S35NC2	1.7	2.3	1.6	2.1
S35NC3	1.2	2.5	1.0	2.4
S35NC4	2.7	5.2	2.5	5.5

## Nomenclature

API	American Petroleum Institute
BWOC	By Weight of Cement
CH	Calcium Hydroxide (Portlandite)
C-S-H	Calcium Silicate Hydrates
OPC	Ordinary Portland Cement
OWC	Oil Well Cement
SF	Silica Flour
SG	Specific Gravity
mD	Millidarcy
NC	Nanoclay
